# The usefulness of quantitative ^99m^Tc-HMPAO WBC SPECT/CT for predicting lower extremity amputation in diabetic foot infection

**DOI:** 10.1038/s41598-024-59764-3

**Published:** 2024-04-22

**Authors:** Soo Bin Park, Chae Hong Lim, Dong-il Chun, Yong Jae Kim, Tae Hyong Kim, Jung Mi Park

**Affiliations:** 1 Department of Nuclear Medicine, Soonchunhyang University Seoul Hospital, Soonchunhyang University College of Medicine, Seoul, Republic of Korea; 2 Department of Orthopedic Surgery, Soonchunhyang University Seoul Hospital, Soonchunhyang University College of Medicine, Seoul, Republic of Korea; 3 Department of Radiology, Soonchunhyang University Seoul Hospital, Soonchunhyang University College of Medicine, Seoul, Republic of Korea; 4 Division of Infectious Diseases, Department of Internal Medicine, Soonchunhyang University Seoul Hospital, Soonchunhyang University College of Medicine, Seoul, Republic of Korea; 5 Department of Nuclear Medicine, Soonchunhyang University Bucheon Hospital, Soonchunhyang University College of Medicine, Bucheon, Gyeonggi Republic of Korea

**Keywords:** Radionuclide imaging, Diabetes complications

## Abstract

We investigated the usefulness of quantitative ^99m^Tc-white blood cell (WBC) single photon emission computed tomography (SPECT)/computed tomography (CT) for predicting lower extremity amputation in diabetic foot infection (DFI). A total of 93 feet of 83 consecutive patients with DFI who underwent WBC SPECT/CT for treatment planning were retrospectively analysed. The clinical and SPECT/CT parameters were collected along with the measurements of the maximum standardized uptake value (SUVmax) at DFI. Statistical logistic regression analysis was performed to explore the predictors of LEA and receiver operating characteristic (ROC) curve was analysed to assess the predictive value of SPECT/CT. The independent predictors of amputation were previous amputation (OR 11.9), numbers of SPECT/CT lesions (OR 2.1), and SUVmax of DFI; either continuous SUVmax (1-increase) (OR 1.3) or categorical SUVmax > 1.1 (OR 21.6). However, the conventional SPECT/CT interpretation failed to predict amputation. In ROC analysis, the SUVmax yielded a fair predictor (area under the curve (AUC) 0.782) of amputation. The model developed from these independent predictors yielded an excellent performance for predicting amputation (AUC 0.873). Quantitative WBC SPECT/CT can provide new information useful for predicting the outcomes and guiding treatment for patients with DFI.

## Introduction

Foot infections are among the serious consequences developed in over half of the cases of diabetic foot ulcers, which are frequent complications of diabetes^1^ Despite advancements in the management of diabetic foot infection (DFI) is facilitated through wound management, vascular assessment, antibiotics, and surgery, DFI remains a major contributor to lower extremity amputation (LEA)^2^. Moreover, the presence of peripheral arterial occlusive disease (PAOD) in diabetic patients complicates management of DFI by impairing blood flow, delaying wound healing, reducing antibiotic efficacy, and increasing the risk of severe complications, including gangrene. As a result, DFI can become incurable or even lead to septic gangrene if not treated promptly and appropriately^3–5^. And the patients may need to undergo LEA to control infection or develop multiorgan failure. Eventually, these patients show a high mortality rate following amputation, ranging from 39 to 80% at 5 years, which makes it urgent to develop an efficient treatment strategy^6^.

For a better outcome of DFI and to avoid the worst adverse consequences, especially amputation, more efforts are needed in predicting the prognosis^6–10^. Unfortunately, clinical findings are somewhat subjective since diabetic patients often have comorbidities, including ischemia and neuropathy, and the systemic signs of inflammation, such as fever and leucocytosis, are often absent even with a serious foot infection^11,12^. Because DFI involving bone portends a worse prognosis than DFI limited to soft tissues, imaging studies have focused on diagnosing osteomyelitis in DFI^13,14^. However, assessing the severity of an infection with imaging studies can be challenging owing to the variable interaction of superimposed infectious and non-infectious processes^15,16^. As such, there is no efficient imaging tool established yet to predict amputation and guide treatment decisions for patients with DFI^17,18^.

^99m^Tc-hexamethylpropyleneamine oxime (HMPAO)-labelled white blood cell (WBC) scintigraphy has proven useful for the diagnosis of DFI^19^. Recently, hybrid single photon emission computed tomography (SPECT) and high-resolution computed tomography (CT) (SPECT/CT) have become particularly important, because they offer significant improvements in both the assessment of local WBC accumulation and provide a detailed depiction of the anatomical bone change. Accordingly, a majority of studies on WBC SPECT/CT have primarily focused on its diagnostic ability for infection and osteomyelitis^20–23^. However, there is limited research on the potential prognostic role of WBC SPECT/CT^24^. While novel SPECT technology can be applied to quantify the degree of radionuclide accumulation by standardized uptake value (SUV)—a well-established prognostic biomarker in oncologic PET—the clinical relevance of measured SUV in WBC SPECT/CT remains unexplored^25–30^. Thus, this study aimed to assess the usefulness of WBC SPECT/CT and SUV in predicting amputation as a prognostic outcome in individuals with DFI.

## Materials and methods

### Subjects

We retrospectively reviewed the medical records of 92 consecutive patients with suspected DFI who visited our multidisciplinary Diabetes Foot Clinic and underwent foot WBC SPECT/CT between October 2016 and July 2020. WBC SPECT/CT was performed to diagnose infection and to evaluate any imaging evidence of osteomyelitis. Among them, the patients who refused to undertake the planned treatment were excluded from further assessment. The patients who underwent amputation or completed treatment with a follow-up of at least 3 months after the end of antibiotic therapy were included in the study (n = 83).

The diabetic foot multidisciplinary clinic comprises podiatrists, orthopedic surgeons, infectious diseases specialists, vascular surgeons, plastic surgeons, endocrinologists, and interventional radiologists. The diagnosis and management of DFI were in accordance with the guidelines established by the international working group on the diabetic foot (IWGDF)^1,2^. The treatment plans, which included medical, surgical, and vascular interventions were decided after comprehensive discussions and collaborations among these specialists. These determinations were based on clinical examinations and imaging findings of one or more study including WBC SPECT/CT, plain radiography and MRI of foot, Doppler ultrasonography and CT angiography of both lower limbs. The decision to perform curative amputation was made on a case-by-case basis by multidisciplinary discussions. Furthermore, several known risk factors were taken into consideration, including the severity of infection, extent of tissue necrosis, presence of peripheral arterial disease, and overall medical condition of the patient. Amputation was considered the last choice for the treatment of non-salvageable limbs when conservative treatments proved ineffective or when the infection posed serious complications such as gangrene, sepsis, and systemic complications threatening the patient’s life.

Soonchunhyang University Seoul Hospital Institutional Review Board approved this retrospective study (No. 2020-07-028) and waived the requirement for written consent. All retrospective analyses involving human participants were performed in accordance with the ethical standards of the institutional and/or national research committee and the principles of the 1964 Declaration of Helsinki and its later amendments or comparable ethical standards.

### ^99m^Tc-HMPAO-WBC SPECT/CT

The labelling of autologous WBCs with prepared ^99m^Tc-HMPAO (Ceretec; GE Healthcare, Oslo, Norway) was based on previously published consensus papers and guidelines with adjustments to the local needs^31^. WBC SPECT/CT was performed on a dedicated hybrid dual-head SPECT/CT scanner (Symbia Intevo, Siemens Medical Solutions, IL) using a low-energy high-resolution, parallel hole collimator 4 h after the intravenous injection of 740 MBq ^99m^Tc-HMPAO-labeled WBCs. SPECT was acquired at 64 stops per detector (angle of 2.813°) at 25 s/stop. CT was performed without using a contrast agent with a 16-slice helical CT scanner, and the images were reconstructed into 0.75-mm slices (130 kV, 100 mA in the Auto-mA mode, and pitch 1.2). CT-based attenuation-corrected SPECT and quantitative SPECT were reconstructed on 256 × 256 matrices using Flash 3D-ordered subset expectation maximization and an ordered subset conjugate gradient maximization (xSPECT Quant, Siemens Medical Solutions, IL) algorithms (eight subsets and four iterations; Gaussian filter of 8.0), respectively. Finally, the SPECT and CT images were co-registered as fusion SPECT/CT images.

### SPECT/CT image analysis

SPECT/CT images were interpreted on an image-analysis workstation (MIM version 6.6, MIM Software Inc., OH) by two board-certified nuclear medicine physicians who were blinded to the clinical results. SPECT/CT was performed to investigate the presence of any active infectious/inflammatory lesion on the diabetic foot, as indicated by an abnormal increase of radioactivity compared to the background. Defects of radioactivity (e.g., cold defects) distal to or at the DFI were considered to indicate gangrene. The number and location of the active lesions were collected. The location of the lesion was determined based on whether the lesion was confined to the forefoot or had affected the midfoot and hindfoot regardless of the forefoot involvement. Conventional binary (osteomyelitis vs. no osteomyelitis) interpretations were performed; bone involvement was defined as the radioactive foci coming in contact with the bone or extending into the marrow space with/without corresponding anatomical bone changes on CT images^20,24^. On the other hand, the radioactive foci were localized only in the soft tissues with no bone involvement. After individual assessment of the images, consensus was reached between the two observers in cases of any disagreement.

For quantitative analyses, the standardized uptake value (SUV) was calculated as the ratio of the radioactivity concentration in voxel measured by the SPECT/CT scanner and the injected radioactivity by the body weight. A spherical volume of interest (VOI) was drawn over the DFI to fully cover it on axial, coronal, and sagittal SPECT/CT images, and the maximum SUV (SUVmax) within the VOI was obtained. The highest SUVmax of the DFIs was collected for each foot. Furthermore, to validate SUV quantification as a relatively new technology, a VOI of 1-cm diameter was placed at the bilateral popliteal veins and the bilateral soleus muscles, and the mean SUV (SUVmean; average SUV of multiple pixels within the VOI) was collected^32^.

### Statistical analysis

The clinical and SPECT/CT variables were compared between patients who underwent LEA during the follow-up period and those who did not undergo amputation using a two-sample t-test, the Mann–Whitney U-test, and the Chi-squared test. Univariable and multivariable logistic regression analyses were performed to evaluate the predictors of LEA. A receiver operating characteristic (ROC) analysis was performed to demonstrate the optimal cutoff value of SUVmax in predicting amputation. The accuracy of the model in predicting amputation was evaluated with the area under the ROC curve (AUC). The interrater reliability test for interobserver agreement of binary interpretation was performed with Cohen’s kappa (κ) statistics. Statistical analyses were performed using MedCalc® Statistical Software version 19.5.2 (MedCalc Software Ltd., Ostend, Belgium), with *P* < 0.05 considered indicative of statistical significance.

## Results

### Patient characteristics

A total of 83 patients (totalling 93 feet) were ultimately included in the study (Table 1). LEA was performed on 50 limbs (53.8%) of 48 patients within the median follow-up period of 391 days (interquartile range, 160–631 days). Six patients died after LEA. A history of LEA was more common among patients who underwent amputation than among those who were not operated (*P* = 0.017). The serum inflammatory markers (e.g., WBC count, ESR, and CRP level) at the time of WBC SPECT/CT were significantly higher in patients who underwent amputation than in those who did not. However, age, sex, comorbid renal impairment, procedure of revascularization, and HbA1c were not significantly different between patients without and with amputation.

**Table 1:** Patient characteristics by the outcome of lower limb amputation.

Clinical variables	Total (n = 83)	Amputation		
		No (n = 35)	Yes (n = 48)	*P*
Age (y)	65.2 ± 12.5	62.2 ± 12.4	67.5 ± 12.2	0.061
Sex, female	23 (28.4%)	11 (31.4%)	12 (26.1%)	0.780
End-stage renal disease	37 (44.6%)	12 (34.3%)	25 (52.1%)	0.165
Revascularization	36 (43.4%)	11 (31.4%)	25 (52.1%)	0.099
Previous amputation	19 (22.9%)	3 (8.6%)	16 (33.3%)	0.017
WBC count (10^9^/L)	7.4 (6.3, 10.4)	7.2 (5.4, 8.2)	8.6 (6.7, 12.4)	0.005
> 10.0	22 (26.5%)	4 (11.4%)	18 (37.5%)	0.016
ESR (mm/h)	91 (56.5, 120)	81 (47.5, 100.5)	107.5 (69.5, 120)	0.020
> 70	56 (67.5%)	20 (57.1%)	36 (75%)	0.140
CRP (mg/dL)	1.5 (0.6, 5.9)	0.8 (0.3, 1.4)	4.2 (0.9, 7.8)	< 0.001
> 0.5	64 (77.1%)	24 (68.6%)	40 (83.3%)	0.188
HbA1c (%)	7.1 (6.2, 8.6)	7.3 (6.4, 9.3)	7.0 (6.2, 7.8)	0.162
≥ 7.5 (58 mmol/mol)	34 (41.0%)	16 (45.7%)	18 (37.5%)	0.599

Parentheses for percentage for categorical variables and interquartile range for continuous variables.

### WBC SPECT/CT results

Foot-based SPECT/CT results were compared between the feet that underwent amputation and those that did not (Table 2). A total of 51.6% of feet presented a single lesion, and the others displayed more than one lesion. The amputated feet displayed more active lesions than the non-amputated feet (median number; 2 vs. 1, *P* = 0.001). The lesions involving the mid- and hindfoot were more frequently observed in the amputated feet than in the non-amputated feet, but without confidence (20% vs. 4.6%, *P* = 0.059). Gangrene, which manifested as a distal cold defect, was observed in 10.8%, all of which were amputated feet (*P* = 0.002). In the conventional binary interpretation (osteomyelitis vs. no osteomyelitis), SPECT/CT evidence of osteomyelitis was recorded in 79.6% of the feet. The osteomyelitis was more frequent in the amputated feet than in the non-amputated feet (90% vs. 67.4%, *P*= 0.015). However, the interobserver agreement of conventional binary interpretation was weak (κ = 0.31, 95% CI 0.00–0.62).

**Table 2:** Results of SPECT/CT analysis of affected foot by the amputation status.

SPECT/CT variables	Total foot (n = 93)	Amputation		
		No (n = 43)	Yes (n = 50)	*P*
Number of lesions	1 (1, 2)	1 (1, 2)	2 (1, 4)	0.001
≥ 2 lesions	45 (48.4%)	15 (34.9%)	30 (60%)	0.027
Location in mid- and hindfoot	12 (12.9%)	2 (4.6%)	10 (20%)	0.059
Distal cold defect	10 (10.8%)	0 (0%)	10 (20%)	0.002
Osteomyelitis	74 (79.6%)	29 (67.4%)	45 (90.0%)	0.015
SUVmax	2.3 (1.1, 5.1)	1.1 (0.7, 2.3)	3.8 (2.1, 5.6)	< 0.001
SUVmax > 1.1	69 (74.2%)	21 (48.8%)	48 (96%)	< 0.001

Parentheses for percentage for categorical variables and interquartile range for continuous variables.

**Figure 1:**
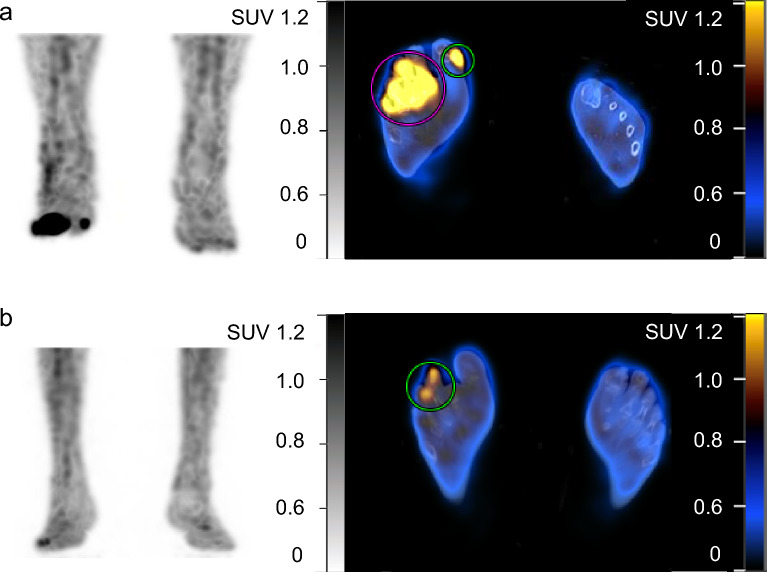
(**a**) Abnormal leukocyte accumulation was observed in the right 1st to 5th toes, with the radioactive lesions extending to the bone, indicating osteomyelitis (left panel, MIP image; right panel, axial SPECT/CT). Two spherical VOIs were drawn on DFIs and the highest SUVmax was 4.2 (higher than the cutoff of 1.1) Despite intensive treatment, the infection progressed, leading to the patient undergoing right below knee amputation 43 days after SPECT/CT. (**b**) Mild focal abnormal leukocyte accumulation was detected in the right 3rd and 4th toes. The lesions were in contact with the bone, which raised a suspicion of osteomyelitis (left panel, MIP image; right panel, axial SPECT/CT). A circular VOI was drawn on DFIs and the SUVmax was 1.0 (lower than the cutoff of 1.1). After 10 weeks of antibiotic treatment, the infection was successfully resolved, and the foot was preserved.

**Figure 2:**
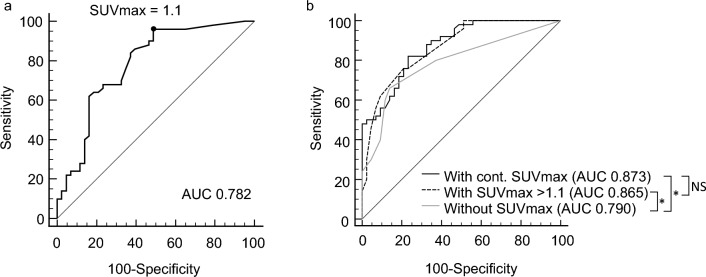
(**a**) The ROC curve illustrates the predictive performance of SUVmax for amputation in patients with DFI (AUC 0.782, 95% CI 0.684–0.861). The optimal SUVmax cutoff was identified as 1.1 (**b**) The ROC curves represent the predictive models derived from independent factors, including history of amputation, number of SPECT/CT lesions, and either continuous SUVmax (AUC 0.873, 95% CI 0.788–0.933) or SUVmax > 1.1 (AUC 0.865, 95% CI 0.778–0.927), or excluding SUVmax (AUC 0.790, 95% CI 0.693–0.868). Cont. indicates continuous, NS indicates not statistically significant; *, *P* < 0.05.

In the quantitative analyses, the amputated feet displayed a higher SUVmax than the non-amputated feet (SUVmax = 3.8 vs. 1.1, *P *< 0.001) (Fig. 1). Moreover, the SUVmax of DFI was higher in the feet with SPECT interpretation of osteomyelitis than in the feet without osteomyelitis (SUVmax = 2.9 vs. 1.1, *P* < 0.001). Using the ROC-derived optimal cutoff for predicting amputation with an SUVmax = 1.1 (Fig. 2a), the amputated feet displayed significantly more lesions with an SUVmax of > 1.1 than did the non-amputated feet (96% vs. 48.8%, *P* < 0.001).

### SUV quantification of the reference vessels and muscles

The SUVmean of the reference vessels (SUVmean = 0.8; interquartile range, 0.6–0.9) and muscles (SUVmean = 0.3; interquartile range, 0.2–0.3) of the bilateral lower legs was analysed in 73 patients with unilateral DFU, excluding 10 patients with bilateral DFI. The quantitative values of the vessels and muscles were not significantly different between the affected and unaffected limbs (vessel SUVmean = 0.8 vs. 0.7, *P* = 0.085 and muscle SUVmean = 0.3 vs. 0.3, *P* = 0.671, respectively). Furthermore, the coefficient of variation of the vessels and muscles was not significantly different between the affected and unaffected limbs.

### Predictors of lower extremity amputation

The results of the univariable and multivariable logistic regression analyses are summarized in Table 3. In the univariable analysis, revascularization (OR 2.9, 95% CI 1.2–7.0), history of amputation (OR 6.3, 95% CI 1.7–23.4), WBC count (OR 1.2, 95% CI 1.1–1.4), ESR (OR 1.0, 95% CI 1.0–1.0), and CRP (OR 1.3, 95% CI 1.1–1.5) were identified as having a prognostic value for amputation. The SPECT/CT parameters were also effective in predicting amputation: the number of lesions (OR 2.0, 95% CI 1.3–3.0), lesions involving the mid- and hindfoot (OR 5.1, 95% CI 1.1–24.9), and SUVmax. Both the continuous SUVmax (OR 1.3, 95% CI 1.1–1.6) and categorical variable with ROC-derived cutoff of SUVmax > 1.1 (OR 25.1, 95% CI 5.4–116.8) were significant predictors of LEA. However, the imaging evidence of osteomyelitis did not act as a significant predictor.

**Table 3:** Predictors for amputation in diabetic foot infection.

	Univariable		Multivariable*	
	OR (95% CI)	*P*	OR (95% CI)	*P*
Clinical variables				
Age (1y-increase)	1.0 (1.0–1.1)	0.102		
Male sex	1.1 (0.4–2.8)	0.836		
End-stage renal disease	2.0 (0.9–4.6)	0.107		
Revascularization	2.9 (1.2–7.0)	0.018		
Previous amputation	6.3 (1.7–23.4)	0.006	11.9 (2.3–61.1)	0.003
WBC count (10^9^/L-increase)	1.2 (1.1–1.4)	0.003		
ESR (1-increase)	1.0 (1.0–1.0)	0.026		
CRP (1-increase)	1.3 (1.1–1.5)	< 0.001		
SPECT/CT variables				
Lesion number (1-increase)	2.0 (1.3–3.0)	0.002	2.1 (1.2–3.8)	0.013
Location in mid- and hindfoot	5.1 (1.1–24.9)	0.043		
Osteomyelitis	1.1 (0.4–2.9)	0.912		
SUVmax (1-increase)	1.3 (1.1–1.6)	0.003	1.3 (1.0–1.5)	0.028
SUVmax > 1.1	25.1 (5.4–116.8)	< 0.001	21.6 (3.6–128.6)^†^	< 0.001

*59.2% Nagelkerke R^2^ using the enter method and adjusted OR for previous amputation, lesion number, and SUVmax; †when SUVmax > 1.1 was substituted for continuous variable of SUVmax, 67.0% Nagelkerke R^2^.

Multivariable logistic regression analysis was performed and corrected for the clinical variables; revascularization, history of amputation, WBC count, ESR, CRP and SPECT/CT parameters; the number of lesions, lesions involving the mid- and hindfoot, and SUVmax. The independent predictors of amputation were as follows: history of amputation (OR 11.9, 95% CI 2.3–61.1), the number of SPECT/CT lesions (OR 2.1, 95% CI 1.2–3.8), and SUVmax; either continuous SUVmax (1-increase) (OR 1.3, 95% CI 1.0–1.5) or categorical SUVmax > 1.1 (OR 21.6, 95% CI 3.6–128.6). When SUVmax > 1.1 were applied instead of the continuous SUVmax in the multivariable logistic regression analysis, the Nagelkerke R^2^ increased from 59.2 to 67.0%. However, when SUVmax was removed from the analysis, the Nagelkerke R^2^ decreased to 51.9%.

In the ROC analysis, the SUVmax resulted fair predictor (AUC 0.782, 95% CI 0.684–0.861) of amputation (Fig. 2a). We developed a predictive model for amputation from these independent predictors (i.e., history of amputation, numbers of SPECT/CT lesions, and continuous SUVmax) achieving an accuracy of 0.873 (95% CI 0.788–0.933) (Fig. 2b). When using the binary variable of SUVmax > 1.1 instead of the continuous SUVmax in the predictive model, the accuracy was not significantly different, with AUC of 0.865 (95% CI 0.778–0.927). However, when SUVmax was removed from the predictive model, the AUC dropped to 0.790 (95% CI 0.693–0.868) (*P* = 0.018).

## Discussion

To the best of our knowledge, this is the first study to investigate the quantitative WBC SPECT/CT as well as to explore the predictive value in patients with suspected DFI. The quantification of leukocyte accumulation was feasible and the SUVmean of the reference muscle was constant, with a median value of 0.3. The SUVmax of the DFI acted as an independent predictor of amputation, showing an adjusted OR of 1.3 (SUVmax 1-increase, 95% CI 1.0–1.5). As the cutoff value of SUVmax increased from > 1.1 to > 12.5, the amputation rate increased from 69.6 to 100%. The ROC-derived optimal cutoff for SUVmax in predicting amputation was determined to be 1.1, demonstrating a high negative predictive value (NPV) of 91.7%. This cutoff proved particularly valuable in predicting favourable outcomes, which aligns with the therapeutic objectives in patients with DFI, that is to prevent amputation.

In leukocyte scintigraphy, injected radiolabelled WBCs migrate to areas of infection or inflammation in the body. This migration, known as chemotaxis, is a biological process in the immune response to infection, where WBCs are recruited to the infection site to combat pathogens and remove damaged cells. Therefore, the intensity of radiotracer uptake at the infection site may reflect the severity of infection. In previous studies and clinical practice, the intensity has been explored qualitatively, comparing it with blood vessel or bone marrow activity, which is subjective and inaccurate to measure^24,33^. Meanwhile, quantitative SPECT/CT techniques have emerged recently, allowing the accumulation of WBCs in the infection site to be quantified as SUV, representing the objective radiotracer intensity. Because the SUV is a degree of accumulated radiolabelled WBCs in the tissues, a higher SUV implies a severe inflammation. Based on the results of our study, we suggest that SUV in WBC SPECT/CT may be useful for evaluating the severity of infection, in line with ability to predict amputation.

Osteomyelitis is a recognized risk factor for amputation in DFI^34^. However, in the present study, conventional binary interpretation (e.g., osteomyelitis vs. no osteomyelitis) failed to predict amputation. In parallel, Erdman et al. reported that the conventional interpretation was less accurate in predicting the therapeutic failure than their scoring system from WBC SPECT/CT based on the multiplicity of lesions, WBC intensity, and the extent of radioactivity^24^. Diagnosing osteomyelitis by SPECT/CT poses challenges due to the interobserver variability and technical limitations associated with hybrid SPECT/CT, including inaccurate spatial registration and the potential impact of patient motion^35^. These factors make it difficult to precisely ascertain whether the detected radioactivity is located in the soft tissues or the bone. However, the SUV is an objective and reliable parameter that can predict LEA in this study.

There were 23 feet (24.7.%) where the prediction by SUVmax cutoff 1.1 differed from the actual clinical outcome. Among the 24 feet with DFI showing SUVmax ≤ 1.1, 2 feet (8.3%) were amputated. One of the amputated feet presented other risk factors, the gangrene and suspicious of osteomyelitis on SPECT/CT, and had a history of previous amputation. For the other amputated foot, the subject was treated with antibiotic treatments and underwent revascularization. However, occlusion of peripheral arteries in the lower legs remained, and the infection progressed to require amputation. Both subjects were suffering from ESRD and undergoing hemodialysis treatments. In contrast, there were 69 feet with DFI presented SUVmax > 1.1, and 21 feet (30.4%) of them were preserved. We performed subgroup analysis for DFI with SUVmax > 1.1 to compare variables by amputation status (Supplementary Table 1). In the non-amputated feet, significantly lower serum WBC count (6.8 10^9^/L vs. 8.7 10^9^/L, *P* = 0.003), lower CRP level (0.8 mg/dL vs. 4.2 mg/dL, *P* = 0.003), and fewer lesions (1 vs. 2, *P *= 0.012) were observed compared to the amputated feet. In fact, the rate of accumulation of labelled WBCs in infection sites is complex and depends on various factors, including not only the virulence and extent of infection but also the type of pathogen, the use of antibiotic or steroid therapy, and the vascular circulation of the infected tissue^33^. Therefore, SUV could serve as a complementary parameter when combined with other clinical information to guide clinical decisions.

We also evaluated several clinical predictors of LEA in patients with DFI. The multivariable analysis revealed that a history of previous amputation acted as an independent predictor of LEA in concordance with previous studies^5,9,34^. However, inflammatory markers were not significant predictors for amputation. The ESR values can be affected by various co-morbidities, and the ESR and CRP levels may not be elevated in acute infections owing to the relatively slow response of these inflammatory biomarkers. Indeed, the role of inflammatory markers is much debated, and although they are usually elevated in infections, they cannot be used to evaluate the severity of infection.

Previous studies have demonstrated predictive models for amputation in patients with DFI using various international classifications or risk scores, and the AUC was 0.67–0.89, which is comparable to our predictive model^36,37^. In this study, the predictive model is relatively simple and easy to compute with only three parameters (i.e., the history of amputation, numbers of SPECT/CT lesions, and SUVmax), making it readily applicable in clinical practice.

The enrolled participants in this study represent a real-world diabetic population with DFI in a university hospital setting. It is important to note that clinical decisions were in accordance with international guidance and based solely on conventional interpretations in routine clinical practice. SUVmax, which was not provided in SPECT/CT reports, did not influence the decision-making process. Hence, we suggest that the criteria for amputation were generally acceptable within the medical field. The limitations of this study include its retrospective design. While our primary aim was to investigate the utility of SUV in predicting the likelihood of amputation, it could also indicate the clinical states that require amputation. We acknowledge the importance of exploring the underlying clinical conditions that may lead to the necessity of amputation and need for a larger prospective study. The predictive model developed based on the available data on our cohort warrants further validation in other populations**.** Moreover, the cutoff of SUVmax should be validated in individual institutions due to the variability of radiopharmaceutical conditions and performance of SPECT/CT scanners.

## Conclusions

The SUV is a simple and reliable prognostic parameter that quantitative WBC SPECT/CT can provide new information useful for predicting the outcomes and guiding treatment for patients with DFI. Based on our experience, patients with DFI exhibiting a higher SUVmax should be treated with more aggressive surgical interventions and prolonged antibiotic treatments when compared with patients exhibiting a lower SUVmax.

### Supplementary Information

Supplementary Table 1.

## Data Availability

The datasets generated during and/or analysed during the current study are available from the corresponding author upon reasonable request.
