# Supplementary material for: The usefulness of quantitative 99mTc-HMPAO WBC SPECT/CT for predicting lower extremity amputation in diabetic foot infection
Source: Sci Rep. 2024 Apr 22;14:9260. doi: 10.1038/s41598-024-59764-3 (PMC11035640; doi:10.1038/s41598-024-59764-3)
Supplement: Supplementary file 1 — Supplementary Table 1. [file 41598_2024_59764_MOESM1_ESM.docx]

Supplementary table 1. Subgroup analysis for DFI with SUVmax >1.1 by amputation status.

|  | Amputation | | |
| --- | --- | --- | --- |
| Clinical variables | No (n = 21) | Yes (n = 58) | *P* |
| Age (y) | 65.9 ± 12.8 | 67.5 ± 11.9 | 0.629 |
| Sex, female | 4 (19.1%) | 12 (25%) | 0.760 |
| End-stage renal disease | 8 (38.1%) | 25 (52.1%) | 0.419 |
| Revascularization | 7 (33.3%) | 25 (52.1%) | 0.240 |
| Previous amputation | 2 (9.5%) | 15 (31.3%) | 0.105 |
| WBC count (10^9^/L) | 6.8 (5.3, 7.7) | 8.7 (6.7, 12.4) | 0.003 |
| > 10.0 | 6 (14.0%) | 19 (38%) | 0.018 |
| ESR (mm/h) | 98 (61, 120) | 107.5 (69.5, 120) | 0.483 |
| > 70 | 26 (60.5%) | 36 (72%) | 0.339 |
| CRP (mg/dL) | 0.8 (0.3, 1.5) | 4.2 (0.9, 8.3) | 0.003 |
| > 0.5 | 24 (68.6%) | 40 (83.3%) | 0.188 |
| HbA1c (%) | 7.3 (6.2, 9.5) | 7.05 (6.2, 7.9) | 0.296 |
| ≥7.5 (58 mmol/mol) | 19 (44.2%) | 20 (40%) | 0.844 |
| SPECT/CT variables |  |  |  |
| Number of lesions | 1 (1, 2) | 2 (1, 4) | 0.012 |
| ≥2 lesions | 15 (34.9%) | 30 (60%) | 0.027 |
| Location in mid- and hindfoot | 1 (4.76%) | 10 (20.8%) | 0.153 |
| Distal cold defect | 0 (0%) | 9 (18.8%) | 0.049 |
| Osteomyelitis | 16 (76.2%) | 44 (91.7%) | 0.119 |
| SUVmax | 2.3 (2, 5.4) | 4.0 (2.1, 5.8) |  |
